# Strain matters: host responses reflect symbiont origin in the squid-vibrio symbiosis

**DOI:** 10.1128/msystems.00498-25

**Published:** 2025-11-17

**Authors:** Vera Beilinson, Grischa Y. Chen, Alexis C. Hargadon, Edward G. Ruby, Margaret J. McFall-Ngai

**Affiliations:** 1 Division of Biology and Biological Engineering, California Institute of Technology 124492 , Pasadena, California, USA; 2 Division of Biosphere Sciences and Engineering, Carnegie Institution for Science, Pasadena, California, USA; Montana State University, Bozeman, Montana, USA

**Keywords:** transcriptomics, development, specificity, squid, *Vibrio*, genomics, diversification

## Abstract

Understanding the cause and consequences of bacterial strain variation remains a challenge in the study of symbioses. While the diverse reactions of the host immune system to strain variants have been well studied in pathogenesis, much less is known about how strain variation influences beneficial associations. From the complex vertebrate gut microbiome to the more tractable invertebrate models of symbiosis, the host’s cellular and molecular responses to this diversity remain largely a mystery. Here, we explore strain diversity in *Vibrio fischeri*, the bioluminescent bacterial symbiont of the Hawaiian bobtail squid, *Euprymna scolopes*. Phylogenetic analyses of the genomes of 62 *V*. *fischeri* strains, including 50 light organ-associated and 12 planktonic isolates, revealed several genes that were absent in planktonic strains, but uniformly present in symbiotic ones. To better understand the consequences of this diversity to the host, we selected five light-organ associated strains: three from *E. scolopes* but having different combinations of colonization factors, one from a congeneric squid host, and one from a marine fish. We colonized juvenile *E. scolopes* with these strains and, using RNAseq, found that (i) the most similar host transcriptomic responses occurred among the native *E. scolopes* strains, (ii) intermediate was the strain from the related squid, and (iii) least similar was the fish strain. Importantly, native strains downregulated immune-related genes more than non-native ones. Finally, host development was atypical or delayed when colonized by non-native strains. These experiments point the way to more targeted studies of the mechanisms underlying host responses to symbiont strain diversity.

**IMPORTANCE:**

Variation among strains of a bacterial species is a powerful factor underlying the intensity of host responses during pathogenic infections. Less is known about the cellular and molecular responses of host tissues to differences between the strains present in an animal's normal microbiome. We use a natural, species-specific, symbiosis to explore the influence of strain-level differences on host gene expression and morphogenesis. Analysis of symbiotic strains from squids and fishes, as well as free-living strains, shows that the carriage of colonization determinants, while critical to competitive success among strains of a species, has a minimal effect on the transcriptional response of the host. We provide evidence that a more important driver of normal gene expression during the development of symbiosis is the history of a strain’s co-diversification with its host species. Such studies, using simple invertebrate models, allow the recognition of otherwise obscured interactions underlying the more complex microbiomes of vertebrates.

## INTRODUCTION

Bacteria exhibit remarkable genome plasticity, enabling the diversification of species into genetically distinct strains that collectively can occupy a wider range of environments and ecological niches. This natural strain-level variation often has profound functional consequences. In host–microbe symbioses of plants and animals, strain-level differences in the bacterial symbiont can shape the success of microbial colonization and persistence, as well as the nature of the host responses to its bacterial partners. For example, differing outcomes of bacterial pathogenesis associated with strain variation has long been known (1), and in some diseases, an individual can carry multiple strains of a pathogen, where such diversity can modulate disease intensity and progression (2). Recent studies have demonstrated that in a mutually beneficial context, natural strain variation appears to be a key feature of the vertebrate microbiome (for review, see references 3, 4). However, because of the bacterial complexity of many microbiomes and the inability to culture many of their component species, defining strain-level characteristics has been challenging although great strides in analysis of this complexity are being made (5).

Several studies using invertebrate models, which typically have a microbiome with fewer species, have demonstrated the importance of strain variation, but few have examined the molecular and cellular impact on the host (see, e.g., 6–9). Naturally occurring monospecific bacterial symbioses offer the opportunity to experimentally manipulate strain variation and study its impact with high resolution. The best studied of such binary associations are the legume–rhizobium symbioses (10), where specificity is dictated by genetic factors from both partners. For example, the bacterial *nodE* gene influences host range by modifying nodulation (Nod)-factor structure, while the host, *Medicago truncatula,* produces nodule-specific cysteine-rich (NCR) peptides that act selectively against incompatible strains (11). Beyond molecular compatibility, functional outcomes also reflect co-evolutionary history. Studies have shown that native rhizobial strains often have a competitive advantage when colonizing the roots of their cognate host, outperforming non-native strains when both are present (12). These findings illustrate a key principle: host-microbe compatibility and potential co-evolution can develop both at the species level and the strain level.

To explore these phenomena within an animal-microbe symbiosis, we have used the species-specific light-organ association between the Hawaiian bobtail squid *Euprymna scolopes* and its bioluminescent bacterial symbiont *Vibrio fischeri* (13, 14). Males of this host have only the *V. fischeri* light-organ (LO) symbiosis throughout their lifespan, whereas females have two sites of symbiosis: the LO, which is present throughout life in both sexes, as well as a consortial accessory nidamental gland (ANG) (Fig. S1), which develops only in mature females (13) and is typical of the females of many squid species. Like the plant-rhizobium association, the binary system of the juvenile *E. scolopes* LO provides a tractable model to study how colonization by different symbiont strains influences the host response.

**Fig 1:**
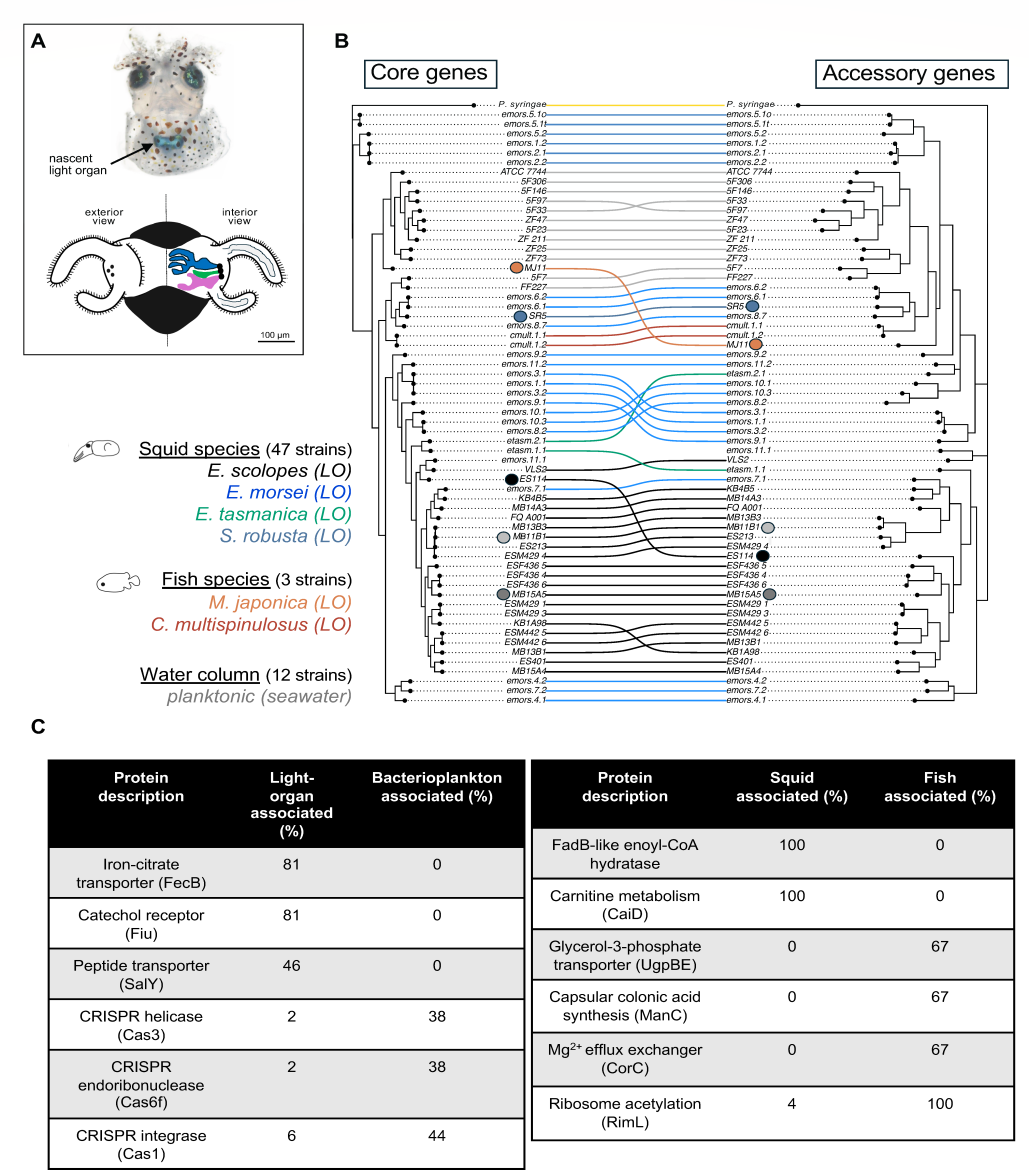
Patterns of gene content in *Vibrio fischeri* isolates diverge across niches and hosts. (**A**) Cartoon of *E. scolopes* nascent light-emitting organ (LO). Left side: external view showing ciliated appendages and three surface pores leading to the internal crypts in which symbionts will be housed. Right side: cut-away view of LO interior showing three of the organ’s six crypts. (**B**) Co-phylogenetic analysis. Left tree: Core genome comparison (nucleotide level) based on orthologous genes. Right tree: Accessory genome clustering based on presence/absence of gene content. Analysis includes 50 light organ-associated strains (from squids or fishes) and 12 planktonic (i.e., seawater) isolates. Trees are rooted on the outgroup species *Pseudomonas syringae*. Branch lengths are log-transformed for visualization. The branch length of *P. syringae* was shortened to fit within the figure. (**C**) Functional enrichment analysis (percent of strains encoding these symbiosis-associated proteins). Left: Proteins enriched in *V. fischeri* LO isolates (50 strains) compared to *V. fischeri* isolates not associated with a LO (12 strains). Right: Proteins enriched in strains associated with squid LOs (47 strains) compared to fish LOs (3 strains).

Juvenile squid hatch into seawater, where *V. fischeri* strains make up <0.1% of the microbial community (15, 16). Nonetheless, it is the only bacterial species capable of entering and colonizing the host’s nascent LO, reflecting the host’s remarkable selectivity for its symbiont. While *V. fischeri* defines the species-level symbiont identity, the LO of the Hawaiian host can be colonized by a number of different *V. fischeri* strains found in the bacterioplankton (17). However, at the level of the individual host, the number of colonizing strains is affected by the biogeography of the organ, i.e., each juvenile host organ has six independent interior crypts and typically entertains between one and six *V. fischeri* strains (Fig. 1A). This observation and other studies have provided evidence that each crypt is usually colonized by a single bacterium (16, 18).

Some strains of *V. fischeri* are not as effective at colonizing the Hawaiian squid host as native strains from *E. scolopes* LOs. For example, MJ11, a *V. fischeri* strain isolated from the LO of the Japanese pinecone fish, *Monocentris japonica*, is unable to colonize *E. scolopes* under typical environmental conditions of low *V. fischeri* abundance (19). MJ11 colonizes poorly because it lacks the gene encoding the Syp-biofilm-promoting regulator, RscS (Fig. S2), which induces the production of an exopolysaccharide that facilitates the colonization of juvenile squid (20, 21). Introducing *rscS* into the MJ11 genome conferred colonization ability under typical conditions, illustrating how specific genetic elements shape host compatibility. These examples highlight how different strains can compete for colonization (14), but how do strain differences affect host responses?

The carriage of several colonization factors differs among native *E. scolopes*-associated strains (14, 22, 23) (Fig. S2). For example, dominant “D” strains are faster colonizers and more competitive than sharing “S” strains, at times excluding them from the crypt spaces (24). Competition within the host crypts, where the symbionts permanently reside, is also reflected in features such as the presence or absence of a type VI secretion system (T6SS) that mediates inter-strain antagonism (25, 26). However, little is known about how carriage of these colonization factors influences host responses.

While much is known about the host’s reactions to colonization by the best-studied *V. fischeri* strain, ES114 (13), the transcriptional and developmental responses to other symbiont strains of different hosts have remained poorly examined. In this study, we compare the effects of colonization by several *V. fischeri* strains, isolated from the LOs of several species of squids and fish, on relative LO gene expression (as based on transcript levels) and developmental phenotypes in juvenile *E. scolopes*. We find that the host’s transcriptional signature varies with the evolutionary and environmental history of the symbiont strain. Specifically, gene-expression responses are most similar when newly hatched juvenile squid are exposed to *E. scolopes* LO isolates, intermediate for strains derived from another species of squid, and most divergent for a fish LO isolate. In addition, several differences in the trajectory of juvenile light-organ development were observed. These findings reveal that the host can discriminate among conspecific strains, suggesting that host–symbiont compatibility and communication can evolve through fine-scale, strain-level co-adaptation. The results add to growing evidence for the importance of considering bacterial diversity below the species level in studies of beneficial symbiosis (27, 28).

## MATERIALS AND METHODS

### Phylogenetic trees, the pangenome, and functional enrichment

Publicly available *V. fischeri* strains with clearly defined isolation sources were downloaded from NCBI in May 2025. RefSeq (GCF) genome assemblies were used. Pangenome analysis was performed using Anvi’o (v8), and functional enrichment between pairwise comparisons was assessed using anvi-compute-functional-enrichment-in-pan.

Orthologs were identified using OrthoFinder (v2.5.5). A binary matrix of gene presence/absence was generated using default parameters, and a nucleotide-based phylogenetic tree was constructed using the -n flag. Multiple-sequence alignment was performed with MAFFT, and gene trees were inferred using IQ-TREE. Co-phylogeny trees were visualized in R using ggtree (v3.12.0) and ape (v5.8-1).

### Sources of *V. fischeri* strains and construction of an *rscS* expression vector

A total of 62 strains of *V. fischeri* were isolated from a number of light organs and seawater samples (Table S1). Particular emphasis was placed on five strains of symbionts (Fig. 1B): ES114, MB11B1, and MB15A5 were isolated from the light organs of adult *E. scolopes* collected in nearshore waters of Oahu, Hawaii, as previously described (29, 30). Strain SR5 was obtained from the light organ of an adult specimen of the related sepiolid squid *Sepiola robusta,* collected off the French Mediterranean coast (31). Strain MJ11 was isolated from the light organ of an adult Japanese pinecone fish, *Monocentris japonica* (20). Construction of the *rscS* expression plasmid, pLMS33, and its empty control vector, pKV69, and their conjugation into MJ11 have been previously described (20, 32).

### Animal colonization by *V. fischeri* strains

Juvenile squid were exposed to bacterial strains within 4 h of hatching in offshore seawater obtained from Scripps Institution of Oceanography, La Jolla, CA. The inoculum was 50,000 CFU/mL, and exposure was for 3 h, unless otherwise noted. This unnaturally high concentration of *V. fischeri* cells (19) was used to limit the possibility that bacterial availability was limiting the inoculation process. Following exposure, animals were rinsed twice with offshore seawater and transferred to individual vials. At 24 h post-inoculation, luminescence was measured, and individuals were classified as colonized when the relative light unit (RLU) level had exceeded the detection limit of the luminometer (>100 RLU). The animals were transferred every 24 h to fresh offshore seawater as described previously (33). Only colonized animals were maintained for analysis at 72 h.

Squid were considered part of the same clutch if their eggs were laid by the same parental pair at the same time and the hatchlings emerged synchronously. Clutch identity was tracked in downstream analyses to account for genetic background effects.

### Determination of symbiont colonization level

At designated time points, individual squid were transferred to 1.5  mL Eppendorf tubes with a minimal volume seawater and anesthetized on ice. Samples were then frozen at −80°C to reduce surface contamination from external bacteria (34). After thawing, squid were homogenized and plated to determine CFU/mL per individual as described previously (33).

### Calculation of light-organ appendage length

At 72 h post-inoculation, squid were preserved on ice in 4% paraformaldehyde (PFA) in marine PBS (mPBS) consisting of 50 mM Na_2_PO_4_, 0.45 M NaCl, pH 7.4 (35). Samples were incubated overnight at 4°C on a rotating platform and then rinsed four times for 15 min with 4  mL mPBS. Squid tissue was stained with the nuclear stain DAPI (Thermo Scientific, Irwindale, CA) diluted 1:50 in mPBS for 24 h, followed by two rinses in mPBS to remove excess stain. Samples were mounted on glass depression slides with coverslips. Imaging was performed on an upright Zeiss (Dublin, CA) LSM 900 microscope. Arm length was estimated in FIJI (ImageJ) using the measurement tool, from a point at the center of the three pores to the tip of each arm.

### Measurement of bottleneck closure

Squid were colonized overnight (18 h) with a single *V. fischeri* strain at an inoculum of 10,000 to 50,000 CFU/mL. Colonization was confirmed using a luminometer; animals with luminescence below ~100 relative light units (RLU) were excluded from analysis to ensure consistent colonization. Squid were anesthetized on ice to prevent premature venting of their symbionts and fixed at 4°C in 4% PFA in mPBS for 24 h with rotation. Samples were rinsed four times for 15 min in mPBS. Light organs were dissected and incubated for 24 h in 400  µL mPBS containing 1% Triton X-100, DAPI (1:50), and rhodamine phalloidin (Biotium, Freemont, CA) diluted 1:50 in mPBS. Samples were rinsed two times in mPBS and mounted on glass depression slides with coverslips. Imaging was performed on a Zeiss LSM 900 confocal microscope. DAPI was used to visualize host nuclei, confirming colonization, and rhodamine phalloidin was used to stain filamentous actin to easily visualize and determine bottleneck diameter, as previously described (36). On both sides of the organ, crypt 1 (Fig. 1A), the most mature crypt with the least variation in structure (37), was imaged, and bottleneck diameters were measured using FIJI.

### Transcriptome assembly, annotation, and differential gene-expression analysis

A previous study had shown that *V. fischeri* colonization by strains isolated from the *E. scolopes* LO significantly changed host gene expression by 24 h post inoculation (hpi) (38). In this study, based on transcript levels, we characterized symbiosis-induced changes in gene expression at 72 hpi to minimize any effects of delayed colonization or morphological development by non-native strains. The CFU and luminescence differences among strains were negligible. At 72 hpi, squid were preserved in RNAlater (Thermo Fisher) overnight at 4°C and stored at 80°C until RNA extraction was performed. Light-organ total RNA was extracted using the Qiagen RNeasy Mini Kit with QIAshredder columns (Qiagen, Valencia, CA) per manufacturer’s instructions. Samples were treated with TURBO DNase (Thermo Fisher) to remove DNA contamination. RNA integrity was assessed using RNA 6000 Pico Kit for Bioanalyzer (Agilent Technologies, Santa Clara, CA). mRNA was separated from total RNA using NEBNect Poly(A) mRNA Magnetic Isolation Module (NEB, Ipswich, MA), and RNA-seq libraries were constructed using the NEBNext Ultra II RNA Library Prep Kit for Ilumina following the manufacturer’s protocols. The libraries were sequenced as paired-end reads (2 × 100 bp) on an Illumina NextSeq2000 at the Millard and Muriel Jacobs Genetics and Genomics Laboratory at Caltech.

Sequencing reads were quality-trimmed using fastp (v0.24.0) with default parameters. Cleaned reads were aligned to the *E. scolopes* reference genome (v2.2) (39) using STAR (v2.7.10b) in two-pass mode with a read overhang of 99. The resulting coordinate-sorted BAM file was used for genome-guided transcriptome assembly with Trinity (v2.1.1), using the --genome_guided_max_intron 500000 flag.

Assembled transcripts were clustered using CD-HIT-EST (v4.8.1) at a 95% similarity threshold (-c 0.95) and then mapped to the genome using GMAP (gmapl, v2024-08-14) with output format gff3_match_cdna. The resulting GFF3 file was converted to BED format using AGAT (v1.4.1), and overlapping features were identified with bedtools intersect (v2.31.0). Novel transcripts were retained and merged with published annotations using AGAT. As is the case for essentially all RNAseq analyses, the annotations reported here are inferred from sequence homologies and are not supported by biochemical confirmation of their functionality. With that understanding, the descriptor “putative” has not been appended in every reference to a geneʻs annotation.

Reads were quantified using featureCounts (v2.0.1) with the updated genome annotation using flags -g gene_id -t exon. Differential expression analysis was performed in R (v4.4.0) using DESeq2 (v1.44.0), with the design formula ~clutch + condition to account for clutch-specific variation. Differential expression between conditions was assessed using contrasts in DESeq2 with Benjamini-Hochberg correction for multiple testing and log2 fold-change shrinkage.

## RESULTS AND DISCUSSION

### Phylogenetic comparisons of free-living and host-associated *V. fischeri* strains reveal a strong clustering by ecological origin

Whole-genome analyses of 62 strains of *V. fischeri* isolated from a variety of sources (Table S1) indicated a strong clustering according to whether they were obtained from seawater (i.e., planktonic) or from the tissues of one of six animal host species (Fig. 1B). Strains obtained from the LOs of *Euprymna* spp. formed distinct clades in the core-genome-based phylogeny (Fig. 1B, left). In general, fish strains, and non-*Euprymna* squid strains clustered separately, suggesting the presence of both host-specific and tissue-specific lineages. This pattern demonstrated that the phylogeny of the *V. fischeri* strains reflects the source of the isolate and supports a possible effect of host-symbiont co-adaptation; however, an influence of geographic distribution is likely also present. Interestingly, analysis using the accessory (or “adaptive”) genome (Fig. 1B, right) revealed a similar pattern, but with higher variation (40), possibly capturing additional strain-level adaptations involving genes that are enriched in isolates from a particular niche. Such a genomic comparison further showed that, while these 62 *V*. *fischeri* strains share a conserved core genome, each strain also encodes a considerable number of singleton genes, i.e., ones that are found in only one strain. An example of such a comparison of singleton gene carriage was depicted for 5 *V*. *fischeri* strains (Fig. S3), showing between 150–400 singletons among those LO isolates. The apparently limited distribution of these singleton genes suggested they have been acquired relatively recently and may be an important class of genetic elements in the diversification of symbiotic *V. fischeri* (17, 41).

Another pattern arising from a genome-level comparison between all host-associated *V. fischeri* strains (Fig. 1C, left) was the high frequency of the carriage of the genes *fecB*, encoding an iron-citrate transporter, and *fiu*, encoding an Fe^3+^-catechol receptor. This pattern was true of strains isolated from both fish and squid LOs. These two Fe-related genes were missing from the genomes of non-LO-associated planktonic strains (Table S1). One interpretation of these results is that strains isolated from host animal tissues encode pathways that support survival in a low-iron environment. Previous research has provided evidence that symbiotic *V. fischeri* is adapted to growth under limiting iron-availability (42, 43), and analysis of the bacterium’s response to colonizing the LO has indicated that, as is true for many animal tissues, the LO is an iron-restricted environment (43–45). However, it should be noted that in some host-associated bacteria, an iron-citrate transporter is used in the opposite direction to efflux excess Fe^3+^ out of the bacterial cell. Reducing the level of free-iron limits the toxic effects of certain host-produced antibiotics and nitric oxide resulting from the Fenton reaction (46). The usefulness of encoding a similar efflux capacity (e.g*., salY*) is also reported for antimicrobial peptides (47, 48). Conditions such as nitric oxide and antimicrobials have been reported in the squid light organ and other host tissues (13, 49), suggesting that the FecB and SalY efflux transporters of *V. fischeri* have a role in the association (Fig. 1C left).

In contrast to LO symbionts, strains isolated as free-living planktonic cells in seawater had a higher likelihood of encoding genes annotated as anti-viral CRISPR-Cas systems compared to host-associated ones (Fig. 1C, left) (Table S2). This difference may reflect the observation that host animals are able to protect their symbiont populations from phage infection while they are in tissues like the LO (50) as opposed to when *V. fischeri* cells are free-living in seawater (51); thus, CRISPR-Cas may be less of a fitness factor for strains that do most of their replication within a symbiotic organ (23). This finding was consistent with the absence of CRISPR-Cas genes in 99% of the sequenced genomes of symbiotic rhizobia, as well as a comprehensive analysis of the patterns of other bacteria lacking CRISPR-Cas (52). Together, these studies, as well as those presented here, have found that symbionts whose growth occurs mainly in their host seem to be more likely to lack CRISPR-Cas. More symbiotic systems should be studied to determine whether loss of CRISPR genes is a general trend among symbiotic species. It is possible that other anti-phage systems (53) may compensate for the loss of CRISPR-Cas in symbiotic *Vibrio* populations. Another consideration is that the loss of CRISPR genes within symbionts may reflect differences in physiological adaptations in the host since CRISPR systems may also function in gene regulation or DNA repair in other bacteria (54, 55).

We also found that the genomes of strains associated with different host species were more likely to encode proteins conferring distinct physiological capabilities (Table S3). For example, as previously reported (22), only isolates from sepiolid squid shared an FadB-like enoyl-CoA hydratase used in lipid degradation and catabolism. Similarly, squid LO-derived *V. fischeri* strains were found to carry the gene annotated as carnitine racemase (*caiD*), used to synthesize osmoprotectants like glycine betaine (56, 57) that could be valuable in conditions of squid tissues, which are typically isosmotic or hyperosmotic to seawater, i.e., at or above 1,000 milliosmoles/liter (mOsm/L) (58) (Fig. 1C, right); however, producing such additional osmolytes would be detrimental to strains growing in the hypoosmotic tissues of marine fishes, which are typically 300–400 mOsm/L (59, 60). Not surprisingly, it has also been reported that the bioluminescence of luminous symbionts isolated from squids requires an osmolarity that is high relative to the optimal conditions of fish LO symbionts (61, 62). Finally, similar to animal pathogens, LO-derived strains of fishes (Fig. 1C, right) more often encoded genes whose annotations are associated with the production of cell-surface modifications (e.g., *manC* and *spsG*) (63, 64) or that lower Mg^2+^ concentration (*corC*) and modify ribosomal components (*rimL*) as a strategy to resist protein-synthesis inhibiting antimicrobials (65, 66). Such immunity-evading mechanisms may provide a higher fitness value for symbionts of a vertebrate host with adaptive immunity. Also notable was a putative high-affinity glycerol-3-phosphate transporter locus (*ugpBE*) that was encoded in fish isolates, but not squid isolates (Fig. 1C). It has been hypothesized that in the squid LO the host provides this metabolite as a nutrient (67) during its daily period of rapid growth (33); however, glycerol-3-phosphate may be less available in fishes, thereby requiring its symbionts to have a higher affinity transporter.

It should be noted that associating certain genetic capabilities with symbiont success in fish LOs (Fig. 1C, right) must be tempered by the low number of isolates from fishes that were analyzed in this study (Table S1). Nonetheless, taken together, the genomic and phylogenetic results presented here provide evidence that *V. fischeri* strains have evolved in response to their ecological context, free-living or host-associated, and that host identity may further drive strain diversification.

### Colonization by different *V. fischeri* strains leads to different transcriptional responses in the host

**Fig 2:**
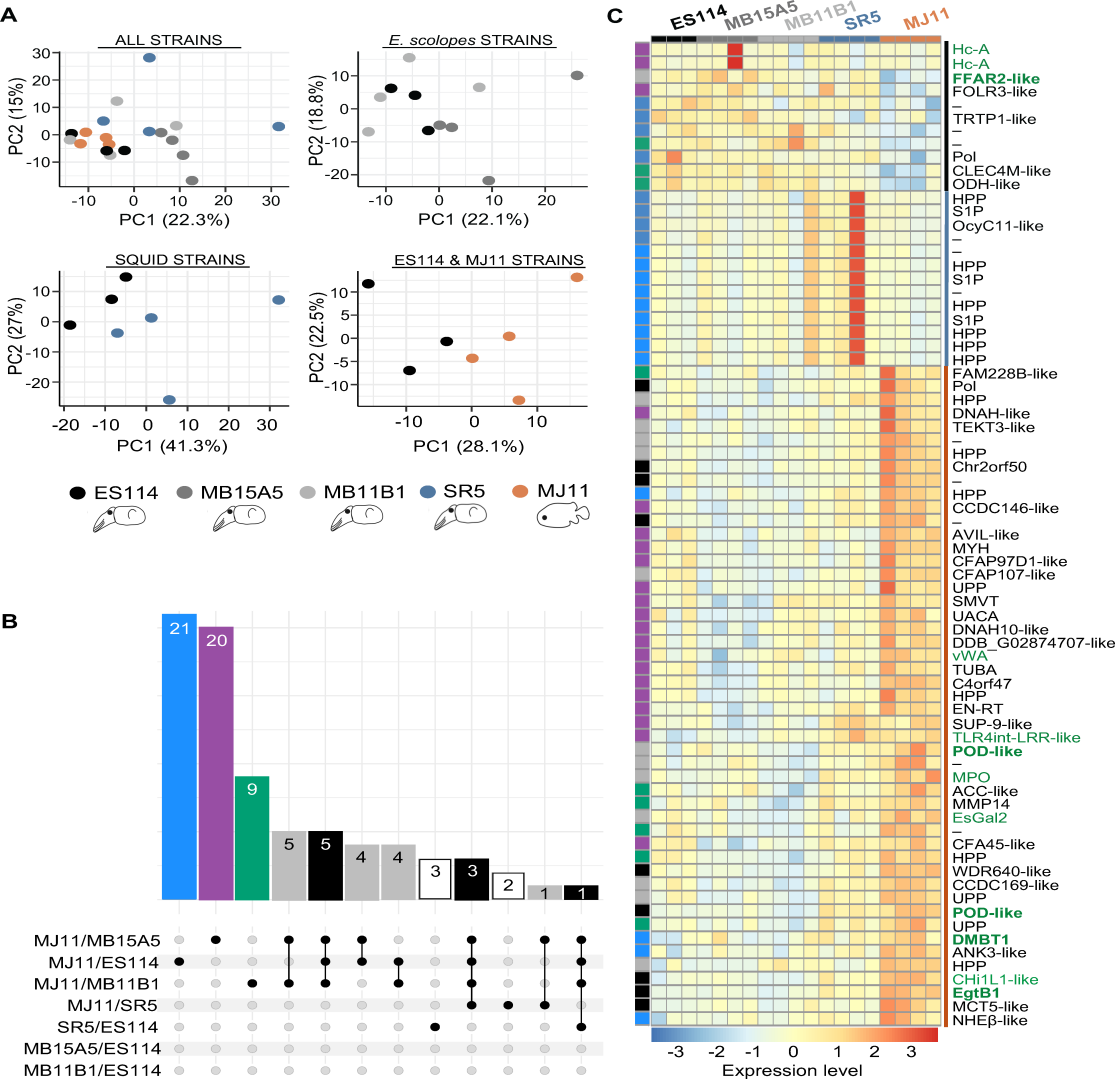
Colonization by non-native *V. fischeri* strains differentially impacts host gene expression at 72 hpi. (**A**) Principal component analysis (PCA) plots representing expression of the top 3,000 most variable host genes 72 h after colonization by one of 5 strains (color-coded). The top-left PCA plot compares all strains to show global variation. The additional three plots highlight differences between specific strain combinations. (**B**) UpSet plot, capturing both shared and strain-specific transcriptional responses, and showing overlap in differentially expressed genes (DEGs) of the host from pairwise comparisons between strains. There were no DEGs between ES114 and the other two *E. scolopes*-derived strains (bottom two rows). DEGs were defined as those with adjusted *P*-value (padj) < 0.05 and absolute log2-fold change (|log2FC|) ≥0.58, with log2FC values shrunken using the *ashr* method. In the MJ11 vs. ES114 comparison, 18 genes had |log2FC| ≥ 1 and 10 had |log2FC| ≥ 2. The 0.58 threshold was selected to capture biologically meaningful changes influenced by bacterial colonization and bioluminescence. Color legend: Blue bar: DEGs unique to MJ11 vs. ES114; Purple bar: DEGs unique to MJ11 vs. MB15A5; Green bar: DEGs unique to MJ11 vs. MB11B1; Gray bar: DEGs in MJ11 vs. one or two other strains (but not all four); Black bar: DEGs shared in MJ11 vs. all *E. scolopes* strains (used in heatmap); White bar: DEGs not included in the heatmap. Color-group memberships of individual DEGs are identified on left column of heat map. (**C**) Heatmap of selected DEGs. Gene annotations are based on BLAST nucleotide hits; annotated gene IDs abbreviated (Table S4); (–) =no BLAST match found; (HPP) = hypothetical/predicted protein; (UPP) = uncharacterized/predicted protein. Functional groupings of GO-term classes are indicated by a colored line on the far right; Black = Transpost and oxidoreductase activity; Teal = Membrane associated proteolysis; Orange = Cytoskeleton, transport and stress response. Gene names with immune-related annotations are in dark green. The five genes examined in Fig. 3 are in bold dark green. Expression values are variance-stabilizing transformed (VST) and row-scaled for visualization. Rows (genes) are clustered by a tree, which is not represented.

In the squid-vibrio field, most research focusing on the host transcriptomic and developmental responses has been done with strains isolated from *E. scolopes*, principally strain ES114 (13, 14). To understand how strain variation influences LO gene expression and development in squid, we colonized newly hatched juvenile *E. scolope*s with strains isolated from LOs across a phylogenetic gradient: (i) ES114, MB15A5, MB11B1 from *E. scolopes*; (ii) SR5 from the Mediterranean sepiolid squid *Sepiola robusta*; and (iii) MJ11 from the fish *M. japonica* (Table S1). Four replicate sets of host colonization were obtained for each strain (Fig. 2C). During a quality control check of the samples, it was determined that one of the four ES114 replicates was an outlier (Fig. S4A). PCA plots of the change in expression of the top 3,000 most variable host genes supported this conclusion (Fig. S4B) so that replicate was eliminated from further analyses.

By 72 hpi, well after colonization had been established and all strains had triggered morphological changes in the LO (13), the overall host RNA profiles remained broadly comparable among strains (Fig. 2A, upper left). However, certain host genes exhibited differences in strain-dependent expression. In subgroup comparisons, the gene expression patterns of the three isolates from *E. scolopes* LOs were similar, forming an overlapping group (Fig. 2A, upper right). In contrast, pairwise comparisons between *E. scolopes* strain ES114 and the non-*E*. *scolopes* LO strains showed distinct groupings (Fig. 2A, lower).

**Fig 3:**
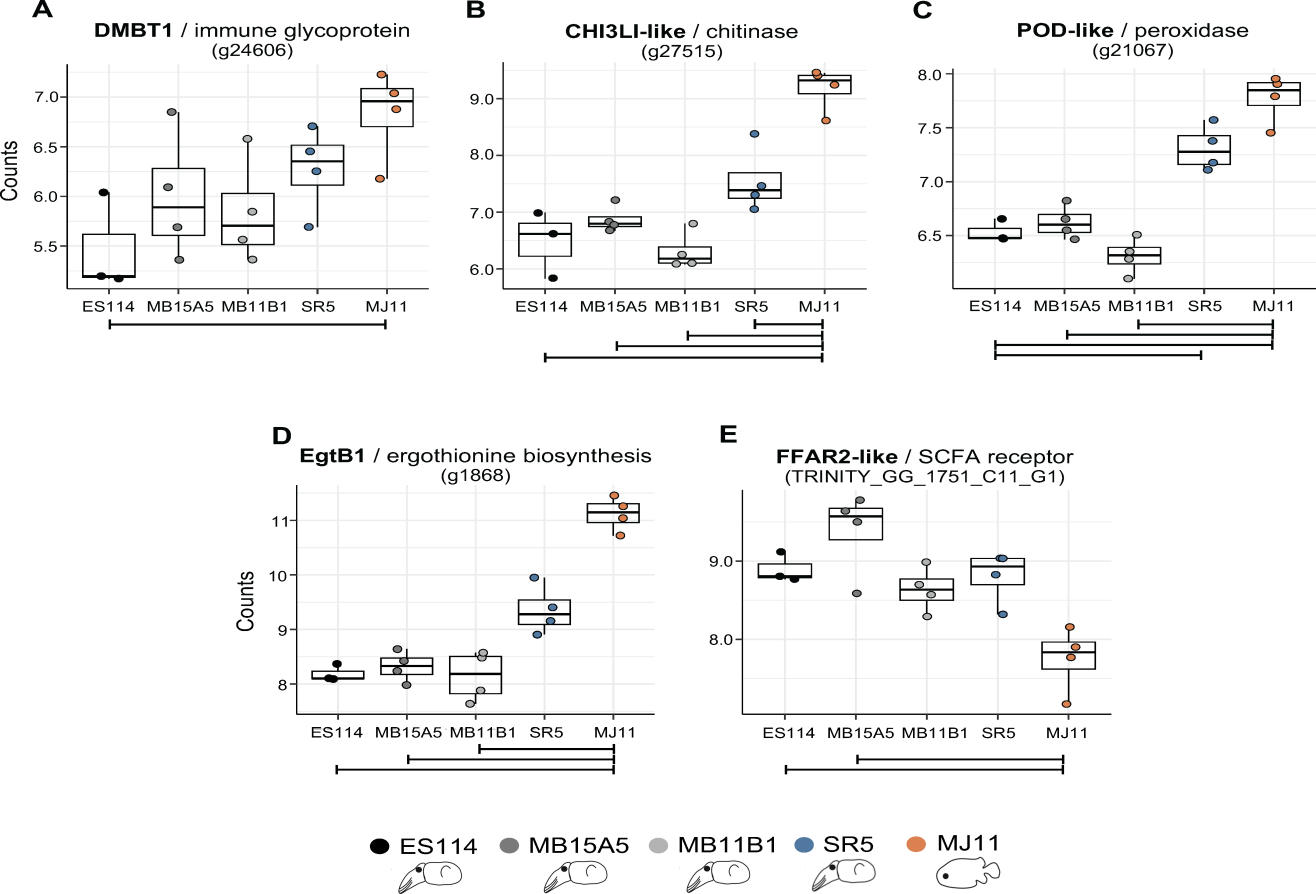
Strain colonization impacts host genes associated with symbiotic interactions. Patterns of expression at 72 hpi of 5 host genes (**A–E**) of interest from Fig. 2C (names in bold). Counts are VST transformed. Expression levels that were significantly different between pairs of strains are indicated by the line below them; padj < 0.05, |log2FC| ± 0.58. For simplicity, SR5 was only compared with ES114 or MJ11. Data are also present in heatmap, Fig. 2C.

We then performed pairwise comparisons between strains with the most distinctive patterns and displayed them as an UpSet plot (Fig. 2B). A major conclusion from this analysis was that the transcriptional response of the host to colonization by any of the 3 *E. scolopes*-derived *V. fischeri* strains was the same (i.e*.,* no DEGs in the bottom two comparisons). The transcription data were also used to develop a heat map displaying differences in transcript level of the 73 most highly regulated genes (Fig. 2C; Table S4). For example, colonization of *E. scolopes* by the fish symbiont MJ11 led to a markedly higher transcript level of a gene predicted to encode glycoprotein DMBT1 than native squid symbionts (Fig. 3A). This protein has a variety of roles in innate immunity, such as the recognition of MAMPs and epithelial-cell differentiation. Several studies have indicated that DMBT1 is a key player in maintaining homeostasis of innate immunity along mucosal surfaces, where it often occurs in high abundance (for review, see reference 68). In a recent contribution, the DMBT1 gene was shown to be regulated in bacteria-associated epithelial tumorigenesis in the mammalian colon (69).

The same pattern of differential regulation was observed with most of the other immune-related proteins (Fig. 2C). For example, a putative antimicrobial chitinase also had higher expression in the non-*E*. *scolopes* isolates (Fig. 3B). Similarly, the genes encoding two proteins associated with the production of antioxidants were differentially regulated among the strains: POD-like, a peroxidase, and EgtB1-like (annotated as “ergothioneine biosynthesis protein 1 isoform 1”). The presence of the gene encoding EgtB1 was unexpected; ergothioneine, a sulfur-containing amino-acid derivative of histidine, is a powerful antioxidant believed to be produced only by certain fungi, actinomycetota, and cyanobacteria (70). When BLASTed against the genomes of the phylum Mollusca, the EgtB1-like mRNA sequence returned a best hit to a sequence encoding a domain predicted to have oxidoreductase activity in the oyster *Crassostrea gigas*. Thus, these data suggest that the EgtB1-like antioxidant protein that, together with the antioxidant POD-like, had significantly lower levels of their encoding transcripts in LOs colonized by strains isolated from *E. scolopes* than by other host-associated strains (Fig. 3C and D). Taken together, these patterns were consistent with previous reports of the native symbiont transcriptionally “calming” the immune response of *E. scolopes* (71, 72), a strategy that non-native *V. fischeri* strains, adapted to other host species, may be unable to deploy.

Finally, some putative immune-related host genes that were differentially regulated when colonized by *E. scolopes*-derived strains, responded less to SR5 or MJ11 (Fig. 2C). For example, the gene encoding FFAR2 (free fatty acid receptor 2) was more highly induced by the native squid strains than by MJ11, the fish LO symbiont (Fig. 3E). In mammals, FFAR2 is a 7-membrane-spanning G-protein-coupled receptor with the highest expression in the gut epithelium, where it senses microbially produced short-chain fatty acids (SCFAs) and acts in concert with other immune system responses to the normal microbiota (73, 74). In *E. scolopes*, the LO symbionts are believed to produce SCFAs that are used by the host (67). It is intriguing to note that the expression pattern seen for the putative FFAR2 is suggestive of a host receptor that is induced only when the native symbiont and its resulting metabolic activity are detected.

Across all comparisons of gene expression, colonization by SR5 usually resulted in an intermediate transcriptional profile, falling between the fish LO symbiont and the three strains isolated from *E. scolopes* LOs (Fig. 3). This trend reinforces the correlation between the nature of host responses to the strain and the relative phylogenetic distance of the hosts from which the symbionts were isolated.

### Colonization by non-native strains alters or delays host phenotypes

**Fig 4:**
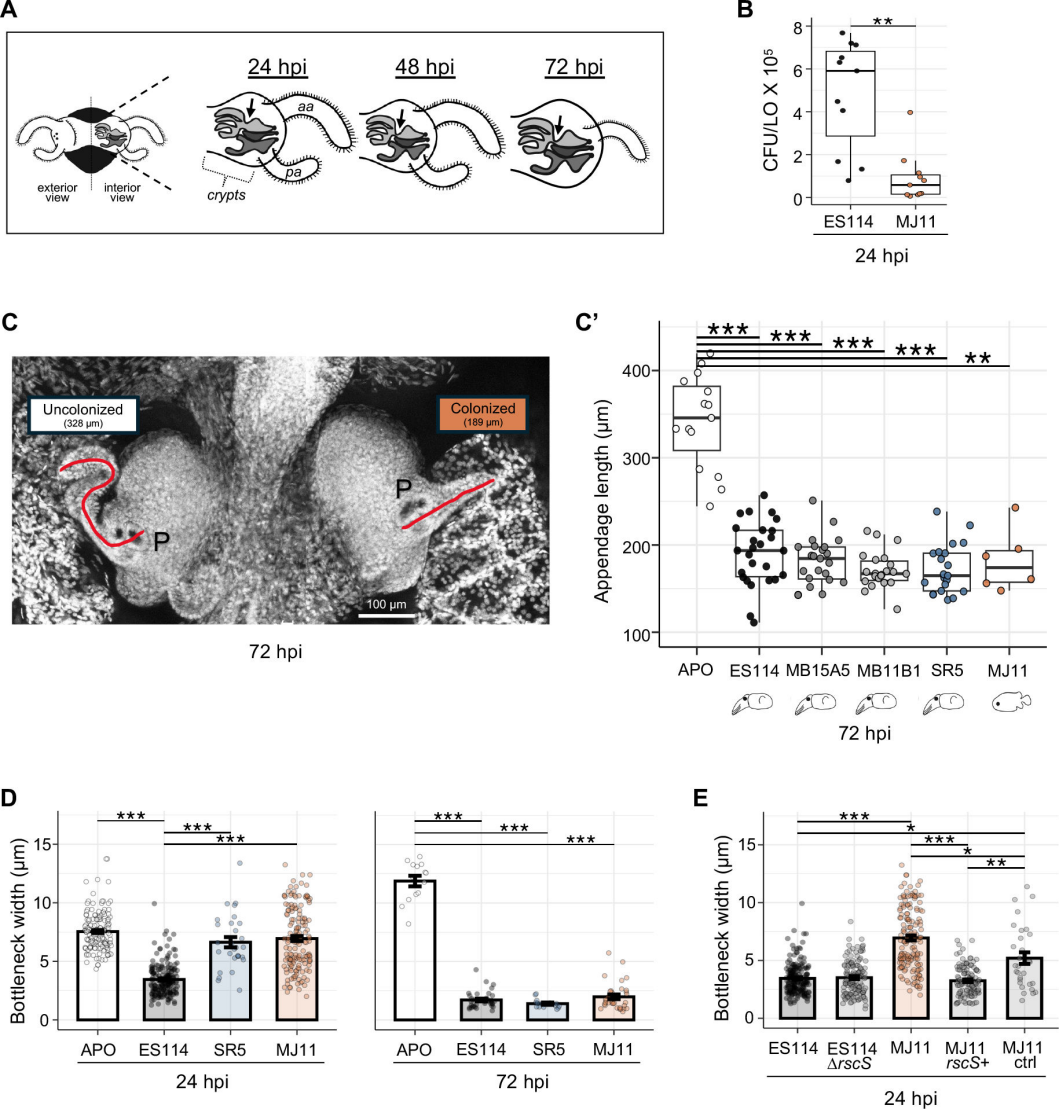
Colonization by non-native strains delays host development. (**A**) Cartoon illustrating some of the developmental events typically occurring in the juvenile LO during the first 72 hpi. The leftmost image shows the entire organ of a hatchling, while the other three depict an interior view of only one side at three times post inoculation. During the first 3 days after colonization, the ciliated appendages begin their trajectory toward full regression, and the bottleneck (arrows) connecting the migration path to the crypts reduces in diameter. *aa*, anterior appendage; *pa*, posterior appendage. (**B**) Colony-forming units per light organ (CFU/LO) recovered from individual squids at 24 hpi. Statistical comparisons were performed using a Kruskal–Wallis test followed by Dunn’s post hoc tests with Bonferroni correction. Asterisks indicate significance: **P < 0.01. (**C**) Image of the LO of a juvenile squid at (72 hpi) colonized on only one side with strain MJ11. This condition was noted in about 25% of MJ11-colonized light organs but was never observed in the several hundred animals colonized by the other four strains. (**C’**) Average length of the ciliated appendages of the LO at 72 hpi with one of five strains. Measurements were made only on animals in which both sides of the organ were colonized. Data shown are for the length of the left anterior appendage. Statistical comparisons were performed using a Kruskal–Wallis test followed by Dunn’s post hoc tests with Bonferroni correction. Asterisks indicate significance: ***P < 0.001. **P < 0.01, * *P* < 0.05. APO (aposymbiotic, not colonized). (**D**) LO bottleneck width at 24 hpi (left) and 72 hpi (right) of colonized LOs compared to uncolonized LOs (APO). Statistical comparisons were performed at each time point, using a Kruskal–Wallis test followed by Dunn’s post hoc tests with Bonferroni correction. Asterisks indicate significance: ***P < 0.001. (**E**) Bottleneck closure response to the carriage of the *rscS* gene. At 24 hpi, bottleneck closure induced by ES114 and ES114 ∆*rscS* was not statistically different; however, MJ11 closed less than both of them. MJ11 *rscS*+ has *rscS* present on a plasmid. MJ11 ctrl carries only the backbone plasmid. Statistical comparisons were performed using a Kruskal–Wallis test followed by Dunn’s post hoc tests with Bonferroni correction. Asterisks indicate significance: ***P < 0.001. **P < 0.01, *P < 0.05. Only some statistical significances are shown for plot clarity.

As mentioned above, in the squid-vibrio field, similar to the study of host transcriptomic responses, most analyses of the host phenotypes have been performed using strains isolated from *E. scolopes*, principally strain ES114 (13, 14). When a host is colonized by strains isolated from squid hosts, both sides of the bilaterally symmetric organ were typically colonized by 24 h and, as such, the symbiont-driven developmental program behaved similarly on either side, as previously reported (13) (Fig. 4A). We found that the fish strain MJ11 initiated symbiosis more slowly and to a lesser extent (Fig. 4B) than any squid-derived strain (23). Specifically, we observed that MJ11 colonized only one side of the LO in 25% of the juvenile hosts, even when exposed to an unnaturally high inoculum, leaving the other half uncolonized for at least 72 hpi (Fig. 4C). This differential colonization was reflected in the morphological development of the organ. While the colonized sides of the organs showed the signature regression of the ciliated superficial epithelium, the sides that were uncolonized had a phenotype similar to the light-organ surfaces of newly hatched (Fig. 1A) or 72 h aposymbiotic animals (75). The appendages of juveniles that were bilaterally colonized by MJ11 or by one of the other strains all regressed to the same degree (Fig. 4C).

We also examined the impact of strain variation on another developmental phenotype (76), the symbiont-induced narrowing of a “bottleneck” that restricts the diameter of the migration path just before the LO crypt entrance (Fig. 4A). This constriction serves to spatially restrict the symbiont population to the crypts, opening only during the crypt venting at dawn (36, 37). By 24-hpi, colonization by *E. scolopes*-derived strains had triggered a significant bottleneck narrowing; however, the non-native strains SR5 and MJ11 had not (Fig. 4D, left). Because bottleneck constriction begins when symbiont numbers are as low as 10 cells (36), the reduced population sizes of these strains at 24 hpi (Fig. 4B) are not responsible for the lack of bottleneck closure. Instead, intrinsic differences between the behavior or biochemistry of native and non-native strains are likely accountable. By 72 hpi, the bottleneck was fully narrowed by all strains (Fig. 4D, right).

To better understand the basis of the strain-level difference in the timing of bottleneck narrowing, we explored how the carriage of *rscS*, which encodes a regulator of biofilm formation in *V. fischeri* (21), may be related to the narrowing of the host’s LO bottleneck (Fig. 4E). The wild-type MJ11 strain, which does not carry *rscS* and colonizes poorly at normal inoculum (20), is delayed in bottleneck constriction at 24 hpi (Fig. 4D, left); however, when strain MJ11 was genetically modified to carry the *rscS* allele*,* colonization resulted in a normal bottleneck narrowing by 24 hpi. Interestingly, the wild-type squid symbiont ES114 typically carries *rscS*, but colonization by a deletion mutant of *rscS* still causes a narrowing of the bottleneck. This result suggested that the carriage of *rscS* is sufficient for bottleneck closure for some strains but is not necessary for others. By 72 hpi, the bottlenecks of all LOs colonized by wild-type and genetically modified strains were closed and not significantly different from strain ES114.  

The bottleneck phenotype is complex, apparently requiring multiple inputs from the symbiont to activate. Our previous work showed that bottleneck closure requires a bioluminescence-independent, quorum sensing-dependent pathway (77). It is also known through multiple studies that quorum sensing and RscS/biofilm formation are transcriptionally and post-transcriptionally co-regulated (78–81). It is possible that in ES114, overlapping signals from both the quorum sensing and biofilm pathways are detected by the host to quickly trigger bottleneck closure. In the absence of any one of these signals, closure may lag or not complete. MJ11 constitutively expressing RscS appears to be primed to express the necessary signals upon colonization of the crypts, whereas wild-type MJ11 takes longer to turn on these signals.

Taken together, the developmental phenotypes described above show that, over time, *E. scolopes* ultimately remodels its LO in response to *V. fischeri* colonization, regardless of the strain’s origin. However, the length of time required for the developmental trajectory to result in normal maturation of the LO varies among *V. fischeri* strains. Similar differences in development have also been found to occur with strain variation in legume-rhizobia associations (10, 11, 82), demonstrating how a comparative strain-level approach can provide clues to symbiotic mechanisms.

### Conclusions and future directions

This study represents a broad view of how symbiont strain-level variation influences the establishment and development of a symbiotic association and serves as a discovery tool for further analysis of this and other symbioses. Specifically, we have shown how host responses to different strains that typically colonize the LO of one host species, *E. scolopes*, differ from the responses of that host to strains from the LOs of other squid and fish species. We anticipate that the patterns in host responses to strain-level differences described here will help generate hypotheses that can be tested in this and other host-microbe symbioses.

The results revealed an array of possible areas in which to more deeply pursue the mechanisms underlying the phenomena discovered. One conspicuous area is the basis for or consequences of changes in gene expression. Specifically, this study did not reveal whether the differences in transcript levels detected during colonization by different strains resulted from changes in the stability of the transcript, or whether the differences were reflected in the levels of transcript-encoded protein abundance and/or activity. Similarly, it remains to be determined whether any of the hypotheses proposed above for underlying functions of the differences noted between strains isolated from squid or fish tissue are valid (Fig. 1C).

Another area of possible exploration would be a comparison with transcriptional responses by other sepiolids species that have symbiotic associations with *V. fischeri* strains. Such an approach would provide insight into whether the responses were common among, or specific to, hosts beyond *E. scolopes*. For example, several *Euprymna* spp. occur in Indo-Pacific and Australian waters, including *E. berryi*, which is being developed as another model sepiolid species (83). In addition, several *Sepiola* spp. with symbiotic organs containing *V. fischeri* occur in the Northeastern Atlantic Ocean and Mediterranean. Such an approach may also allow a refined analysis of the relationship between bacteria genomic differences, which are extensive, and host responses.

## Data Availability

The data supporting the findings of this study are available within the article and its supplemental information files. The transcriptomic raw sequencing data generated in this study have been deposited in Gene Expression Omnibus under accession number GSE306269. Relevant code for this manuscript is available at https://github.com/verabeil/escolopes_vfischeri_strain.

## SUPPLEMENTAL MATERIAL

Supplemental Figures msystems.00498-25-s0001.pdf Figures S1-S4.

Table S1 msystems.00498-25-s0002.xlsx *V. fischeri* strains used in this study.

Table S2 msystems.00498-25-s0003.xls Genomics comparison of LO and non-LO isolates.

Table S3 msystems.00498-25-s0004.xls Genomic comparison of squid and fish isolates.

Table S4 msystems.00498-25-s0005.xlsx Gene IDs and abbreviations used in the text.

ASM does not own the copyrights to Supplemental Material that may be linked to, or accessed through, an article. The authors have granted ASM a non-exclusive, world-wide license to publish the Supplemental Material files. Please contact the corresponding author directly for reuse.
