# Supplementary material for: Strain matters: host responses reflect symbiont origin in the squid-vibrio symbiosis
Source: mSystems. 2025 Nov 17;10(12):e00498-25. doi: 10.1128/msystems.00498-25 (PMC12710313; doi:10.1128/msystems.00498-25)
Supplement: Supplemental Figures — Figures S1-S4. [file msystems.00498-25-s0001.pdf]

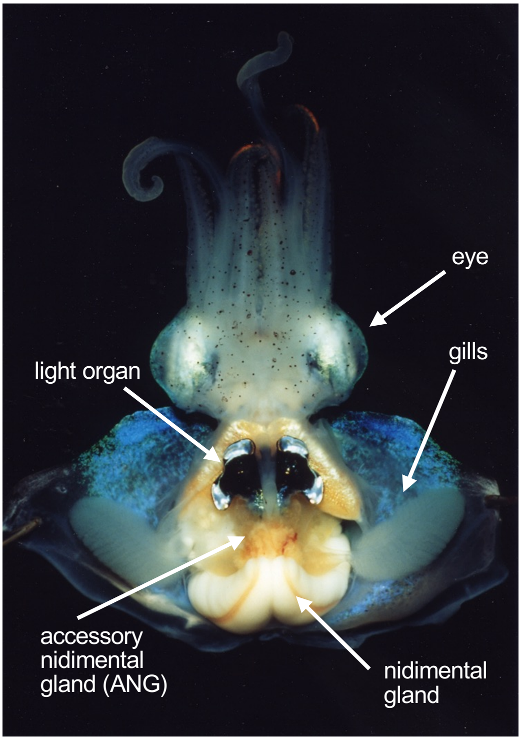

FIG S1 Ventral dissection of a sexually mature adult female *Euprymna scolopes.* The locations of several organs are indicated, including the two containing long-term bacterial symbionts: the light organ and the accessory nidamental gland.

Factors	Colonization stage	Reference
RscS capsule regulator	Initiation (aggregation)	Mandel *et al.* 2009
D-strain dominance behavior	Initiation (motility)	Bongrand *et al.* 2016
T6SS-2 weapon	Persistence	Suria *et al.* 2022

Light-organ symbiont strain	Host species isolated from	Factor encoded		
		RscS	D strain	T6SS-2
ES114 (native)	*E. scolopes*	+	-	-
MB15A5 (native)	*E. scolopes*	+	-	+
MB11B1 (native)	*E. scolopes*	+	+	+
SR5 (non-native)	*S. robusta*	-	-	-
MJ11 (non-native)	*M. japonica*	-	-	+

FIG S2 Factors providing a fitness benefit to *E. scolopes* native LO strains at different stages of colonization are either present (+) or absent (-) in the 5 native and non-native LO symbionts examined for transcriptional and developmental effects on juvenile *E. scolopes*.

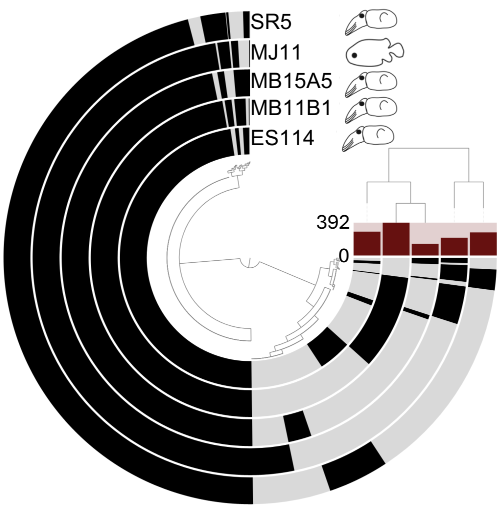

FIG S3 The pangenome of five LO-symbiont strains used in this study. Black lines are genes present in each genome. The dendrogram is built from pairwise average nucleotide identity (ANI). *E. scolopes* symbionts (ES114, MB11B1 and MB13B2) share more similar nucleotides sequences. MJ11 and SR5 are more like each other, and divergent from the *E. scolopes* symbionts. Maroon bar graph indicates number of singleton genes found in each strain.

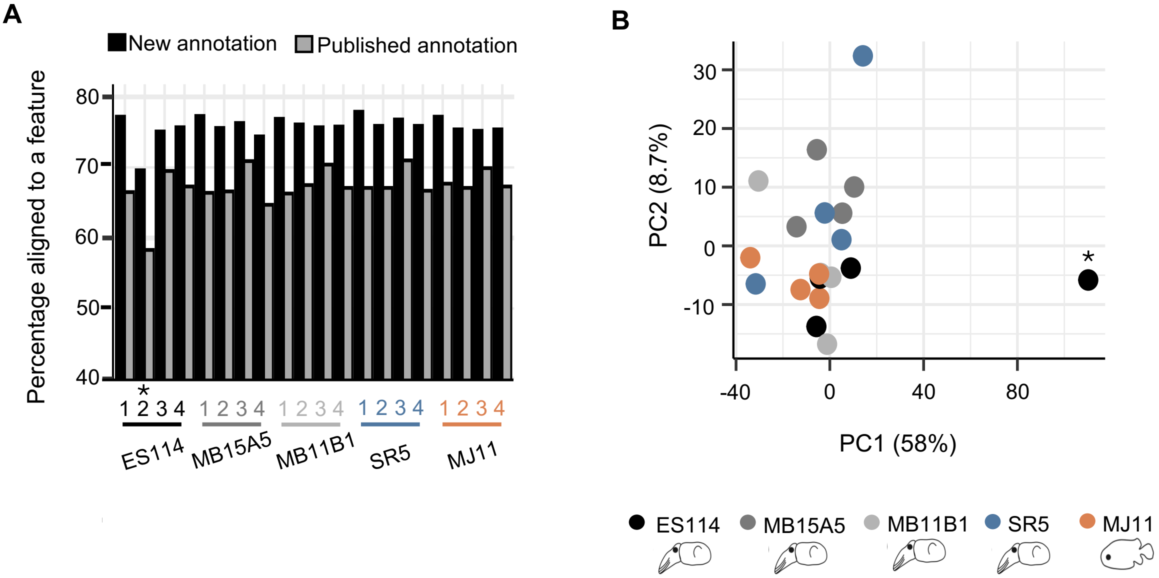

FIG S4 Identification of one experimental replicate as an outlier. (A) Summary of the efficiency of alignments of each replicate to genomic features. (B) PCA plot based on the top 3,000 most variable genes across all samples. ES114 replicate #2, identified as an outlier, was excluded from further analysis. An asterisk (*) indicates the low-quality sample that was removed from downstream analysis.
